# Aryl Hydrocarbon Receptor Establishes a Delicate Balance between the Level of the Trace Amine Tryptamine and Monoamine Oxidase Activity in the Brain and Periphery in Health and Conditions such as Neurodegenerative, Neurodevelopmental, and Psychiatric Disorders

**DOI:** 10.2174/011570159X340635241022113450

**Published:** 2025-01-13

**Authors:** Marta Kot

**Affiliations:** 1 Department of Stem Cell Bioengineering, Mossakowski Medical Research Institute, Polish Academy of Sciences, Pawinskiego 5 Str, 02-106 Warsaw, Poland

**Keywords:** Aryl hydrocarbon receptor (AHR), tryptamine, monoamine oxidase (MAO), brain, gut microbiota, miRNA, CYP enzyme, neurodegenerative, neurodevelopmental, psychiatric disorders

## Abstract

The purpose of this review was to analyse the literature regarding the correlation between the level of tryptamine, aryl hydrocarbon receptor (AHR) signalling pathway activation, and monoamine oxidase (MAO)-A and MAO-B activity in health and conditions such as neurodegenerative, neurodevelopmental, and psychiatric disorders. Tryptamine is generated through the decarboxylation of tryptophan by aromatic amino acid decarboxylase (AADC) in the central nervous system (CNS), peripheral nervous system (PNS), endocrine system, and gut bacteria. Organ-specific metabolism of tryptamine, which is mediated by different MAO isoforms, causes this trace amine to have different pharmacokinetics between the brain and periphery. Reactive oxygen species (ROS) generated by MAO can influence miRNA-CYP enzyme regulatory network and affect mitochondrial function. Tryptamine regulates AHR function by acting as an endogenous ligand for AHR, initiating AHR activation and inhibiting the expression of the CYP1A1 and CYP1A2 genes. The dysregulation of AHR signalling, triggered by endogenous tryptamine binding, can disrupt the regulation of prolactin levels. Depending on the tryptamine concentration and context, tryptamine can be beneficial or harmful. By acting as an agonist of inhibitory serotonin receptors and trace-amine associated receptor 1 (TAAR1) and an antagonist of excitatory serotonin receptors, it can engage in diverse physiological interactions with serotonin. Increased tryptamine production is observed under hypoxic conditions and is associated with hypoxia-inducible factor 1α (HIF-1α) activation, leading to AHR activation. Dysregulation of the association between tryptamine levels, AHR signalling pathway activation, and MAO activity is observed in Alzheimer’s disease (AD), Parkinson’s disease (PD), autism spectrum disorder (ASD) and schizophrenia.

## 1 INTRODUCTION

Tryptophan is an essential amino acid that must be obtained through the diet. Tryptophan metabolism in mammals is a complex and dynamic system consisting of three main interconnected pathways: the kynurenine, serotonergic and indole pathways. The dynamic nature of tryptophan metabolism is visible in the following aspects: changes in the activity of one pathway can affect substrate availability for others; metabolites from one pathway can influence the activity of enzymes in another; diet, stress, and gut microbiota composition can all impact the balance between these pathways and influence the diurnal variations in tryptophan metabolism, as in the case of the serotonergic pathway. Thus, the balance between these pathways is delicate and can be disrupted in various pathological conditions.

The kynurenine and serotonergic pathways are the most extensively analysed routes for tryptophan metabolism. Both pathways have significant implications for various physiological and pathological processes. The indole pathway, while often overlooked, also plays a crucial role in tryptophan metabolism since it involves the conversion of tryptophan into several metabolites, including tryptamine. Tryptamine plays a significant role in the gut microbiome, and abnormal tryptamine levels in humans are linked to various neuropsychiatric, neurodegenerative and neurodevelopmental disorders. Some tryptamine-based drugs, such as sumatriptan, are effective in alleviating migraine symptoms.

The aryl hydrocarbon receptor (AHR) is a ligand-activated transcription factor that plays a significant role in regulating the expression of genes involved in xenobiotic metabolism, immune responses, and neurodevelopment. Its interaction with tryptamine and other metabolites derived from tryptophan can have significant implications for neurodegenerative, neurodevelopmental, and psychiatric disorders.

Monoamine oxidases A and B (MAO-A and MAO-B) play significant roles in the pathophysiology of various neurodegenerative, neurodevelopmental, and psychiatric disorders. These enzymes are responsible for the oxidative deamination of tryptamine, leading to the formation of indole-3-acetaldehyde. This metabolic conversion is essential for the activation of AHR by tryptamine, highlighting the interplay between MAO activity and AHR signalling.

This article aims to analyse the correlations among trace amine tryptamine levels; the activation of the aryl hydrocarbon receptor (AHR) signalling pathway; and the activity of monoamine oxidases (MAO-A and MAO-B) in both healthy individuals and individuals with neurodegenerative, neurodevelopmental, and psychiatric disorders. This article advocates for expanded research into the dynamic relationship between tryptamine levels, activation of the aryl hydrocarbon receptor (AHR) signalling pathway, and MAO enzyme activity, which is an intriguing and promising research direction for neurogenerative, neurodevelopmental and psychiatric disorders. This association is especially notable because it centres on the crucial point where physiological processes shift towards the pathological manifestations observed in Alzheimer’s disease (AD), Parkinson’s disease (PD), autism spectrum disorder (ASD) and schizophrenia.

Understanding the complex relationships among tryptamine levels, MAO enzyme activity and AHR signalling pathway activation in neurodegenerative, neurodevelopmental, and psychiatric contexts can provide valuable insights into the pathogenesis of these conditions, as well as potential therapeutic targets and strategies for their management.

## 2 TRYPTAMINE IN THE BRAIN AND PERIPHERY

**Fig. (1):**
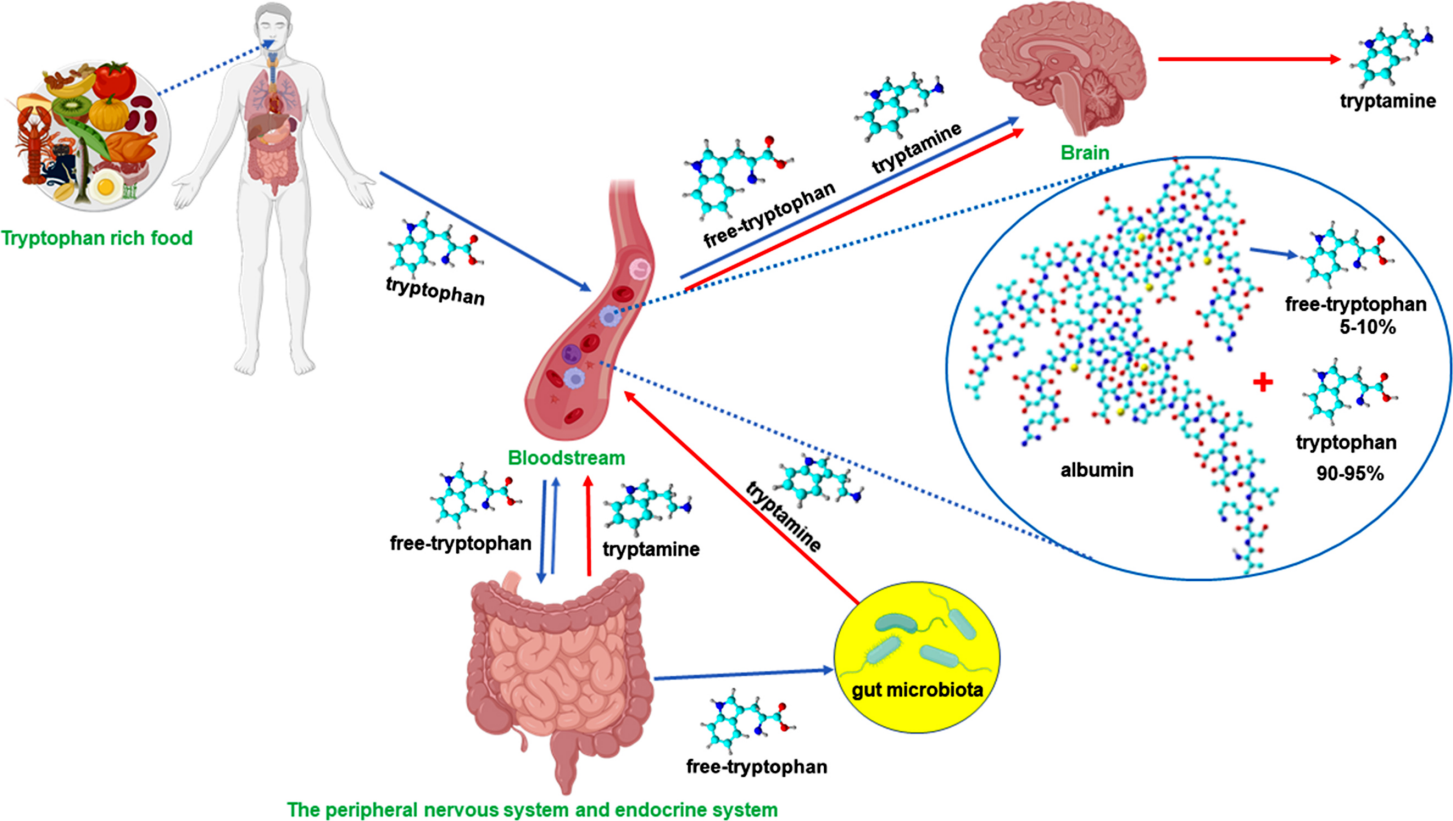
Circulation of tryptamine throughout the body.

An *in vivo* experiment confirmed that the production of tryptamine in the brain is directly influenced by the concentrations of tryptophan in both the brain and plasma [1]. However, after dietary tryptophan is absorbed in the gut and reaches the bloodstream, the majority of it binds to albumin, with a smaller portion (5-10%) remaining unbound [2]. This unbound tryptophan can carry out its functions within tissues [2]. Thus, the availability of tryptophan is controlled by albumin, a fast-acting peripheral factor that is controlled by complex interactions among the immune, endocrine, and serotonergic systems [3]. The coordination of these multidirectional interactions among the immune, endocrine, and serotonergic systems appears to play a crucial role in regulating hippocampal neurogenesis [4]. This finding prompted us to ask whether the binding of tryptophan to albumin is equally critical for regulating the levels of tryptamine in both the brain and the periphery (Fig. **1**). Although tryptamine is present in the central nervous system (CNS) in relatively small quantities (ng/g) [5], the concentration of tryptamine in the blood serum reaches 1600 µg/litre (approximately 10 µM) [6]. This finding suggests that the availability of unbound tryptophan, which serves as a precursor to tryptamine, is also critical for sustaining optimal levels of tryptamine in both the brain and plasma. The successful use of tryptamine as a ligand for human serum albumin (HSA) purification strongly suggests that tryptamine can indeed interact with HSA through mechanisms such as hydrophobic interactions, hydrogen bonding, and potentially other noncovalent bonds [7]. Nevertheless, further research is needed on this topic.

Tryptamine is a naturally occurring compound derived from the amino acid tryptophan, that shares structural similarities with serotonin, also known as 5-hydroxytryptamine (5-HT). Therefore, the physiological association between the function of the serotonergic system and the tryptamine concentration has behavioural implications. In Jones's research, one of the initial observations concerning the role of the relationship between the serotonergic system and tryptamine in behaviour was documented [8]. Intravenous administration of 5-HT or noradrenaline led to a dose-dependent sleep-like sedative effect, while elevated doses of tryptamine induced arousal. In turn, the administration of tryptamine to mice previously treated with an MAO inhibitor resulted in the inhibition of head twitches, representing psychedelic experiences in humans induced by the administration of the serotonin precursor 5-hydroxytryptophan. This change was not observed in the mice pretreated with tryptamine alone. Moreover, tryptamine also reduced swallowing induced by 5-hydroxytryptophan; fluoxetine, a 5-HT uptake blocker, and p-chloroamphetamine (PCA, 2.5 µg/kg), a compound that induces the release of serotonin from presynaptic neurons into the synaptic cleft, resulting in elevated serotonin levels in the brain [8]. These contrasting associations between serotonin and tryptamine levels may underlie the psychoactive effects of PCA, 5-hydroxytryptophan and fluoxetine, as well as their potential neurotoxicity. Notably, the intraperitoneal injection of a high (neurotoxic) dose of PCA (10 mg/kg) leads to a notable reduction in the levels of serotonin and its metabolite 5-hydroxyindoleacetic acid (5-HIAA) across various brain regions, including the cortex, striatum, hippocampus, hypothalamus, cerebellum, and brainstem [9]. Comparable decreases in the levels of serotonin and its metabolite 5-HIAA across brain structures are observed following a tryptophan-free diet [10]. In conclusion, these observations indicate that serotonin and tryptamine levels are inversely correlated.

The tryptamine concentration in the whole brains of normal rats under physiological conditions was found to be 0.60 ± 0.06 ng/g of tissue using high-performance liquid chromatography (HPLC) with fluorescence detection [11]. L-tryptophan administration increased the tryptamine concentration in the brain to 96.7 ± 21.9 ng/g when p-chlorophenylalanine (PCPA, a selective and irreversible inhibitor of tryptophan hydroxylase) was administered intraperitoneally prior to tryptophan [11]. Notably, the intraperitoneal administration of PCPA also caused a significant decrease in the levels of serotonin and its metabolite 5-HIAA within the cortex, striatum, hippocampus, hypothalamus, cerebellum and brainstem [9]. Similar reductions in the levels of serotonin and its metabolite 5-HIAA within brain structures were observed following a tryptophan-free diet [10]. Thus, the physiological relationship between serotonergic system function and the tryptamine concentration depends on a precise balance of tryptamine and serotonin concentrations within the regions in which they function. This physiological association may be the cornerstone of thermoregulatory mechanisms, especially in addiction treatment and when psychoactive substances are used to treat conditions such as posttraumatic stress disorder (PTSD). Cox's research demonstrated that unilateral intrahypothalamic injection of 5-HT resulted in a dose-dependent decrease in core temperature in rats. Conversely, the injection of tryptamine into the same site led to a dose-dependent increase in core temperature [12].

Crucially, the binding sites for tryptamine do not consistently overlap with all serotonin binding sites. More than 40% of neurons in the brain exhibit diverse responses to both amines, serotonin and tryptamine [13]. On the basis of the findings of Jones and Boulton [14], Burchett proposed that tryptamine acts as an agonist of inhibitory serotonin receptors and behaves as an antagonist of excitatory serotonin receptors [15]. Additionally, the inhibitory effects of tryptamine are more potent, occur more quickly, and last longer than those of serotonin.

In addition to having a high affinity for specific serotonin receptors, tryptamine is an endogenous trace amine capable of activating trace amine-associated receptor 1 (TAAR1). TAAR1 is a G protein-coupled receptor primarily found in brain regions associated with the modulation of monoaminergic neurotransmission. This receptor is associated with cyclic adenosine monophosphate (cAMP) signalling pathways. The ability of tryptamine to induce cAMP generation was observed in HEK293 cells with stable expression of rat TAAR1 (TA1) [16], as well as in COS-7 cells transfected with human TAAR1 [17]. However, the response was markedly reduced (half maximal effective concentration (EC_50_)˃6 µM) upon TAAR1 activation by tryptamine [17]. Given that the EC_50_ of tryptamine for TAAR1 is within the micromolar range, whereas typical brain concentrations of tryptamine under physiological conditions are in the nanomolar range, the activation of this receptor by tryptamine may occur only under pathological conditions in which tryptamine concentrations are substantially elevated. Indeed, recent findings have demonstrated that TAAR1 in the dentate gyrus (DG) plays a pivotal role in regulating the detrimental effects of chronic stress on hippocampal plasticity and cognition [18]. Therefore, agonists targeting TAAR1 have therapeutic potential for treating cognitive deficits and metabolic dysregulation linked with major depressive disorder (MDD) and schizophrenia [19]. However, tryptamine can enter neurons independently of 5-HT-transporters, eliciting the release of 5-HT and noradrenaline (NE) from the enteric nervous system [20]. Both 5-HT and NE are associated with gastrointestinal discomfort, nausea, vomiting, and diarrhoea. These adverse events have been noted following the administration of ulotaront (SEP-363856), a pioneering CNS-active substance [21-23]. It functions as an agonist of both TAAR1 and 5-HT1A receptors [21]. Ulotaront is currently undergoing phase 3 clinical trials for the treatment of schizophrenia [22]. Likewise, these adverse effects have been observed during 3,4-methylenedioxymethamphetamine (MDMA; “ecstasy”)-assisted therapy, especially in the management of severe PTSD [24]. Animal experiments have confirmed that amphetamines, including MDMA, are potent TAAR1 agonists [16]. In short, tryptamine is a neuromodulator with unique receptor affinity. Focusing on tryptamine-dependent receptors could lead to therapies that improve cognitive function in patients with MDD and schizophrenia.

**Fig. (2):**
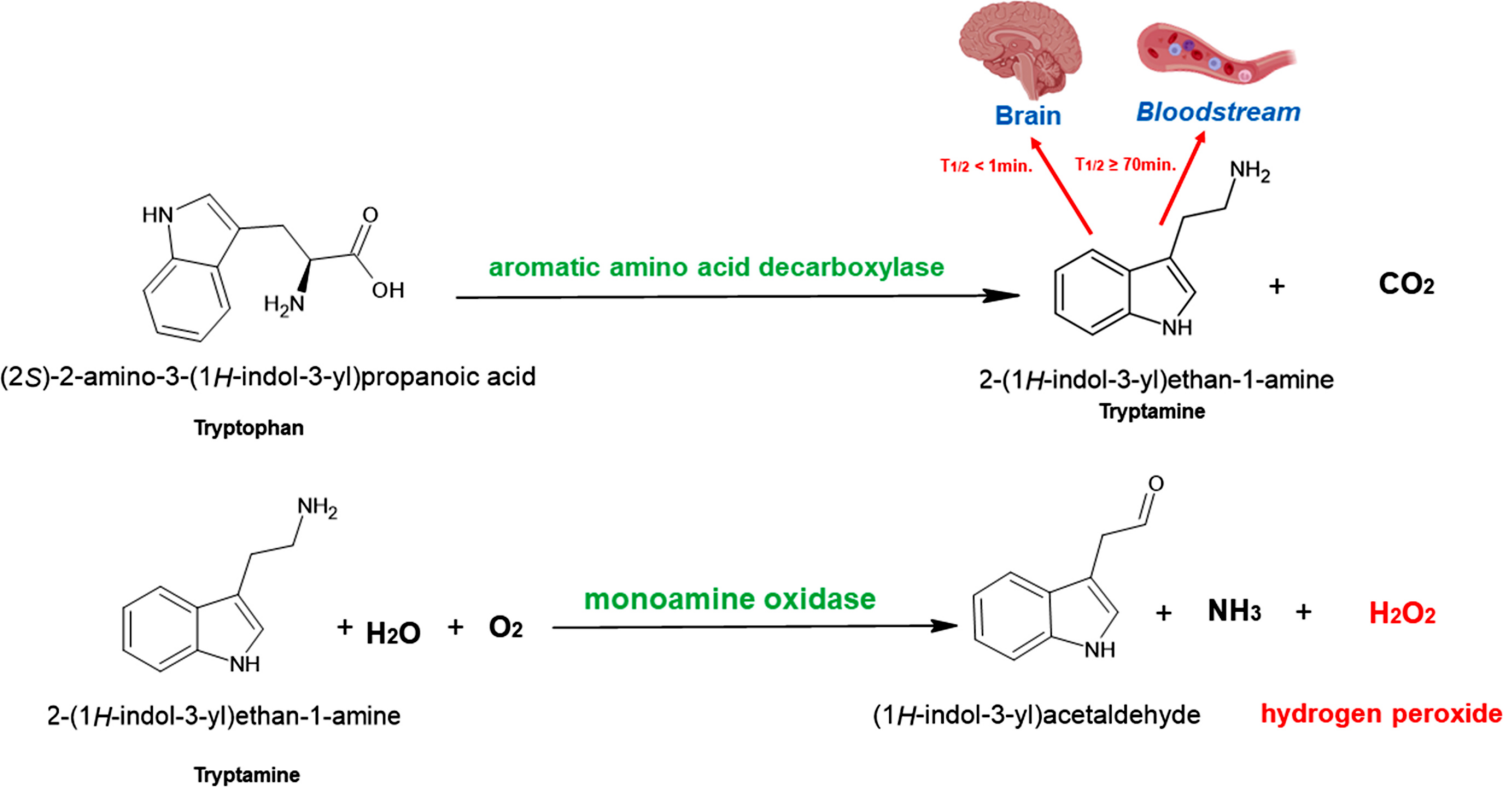
Generation and metabolism of tryptamine in the brain and peripherally.

Direct decarboxylation of L-tryptophan by aromatic amino acid decarboxylase (AADC) is the only process through which tryptamine is produced in the brain (Fig. **2**). Consequently, the generation of tryptamine depends on the activity of L-aromatic acid decarboxylase (L-AADC), which is abundant in monoamine-containing neurons and glial cells [25-28]. However, this enzyme is widely distributed throughout the CNS, peripheral nervous system (PNS), and endocrine system [29]. Moreover, L-AADC exhibits broad substrate selectivity, participating in various processes, including the synthesis of 5-HT from 5-hydroxytryptophan, and serves as the sole enzyme in the synthesis of tryptamine from tryptophan [30]. As a result, altering this enzyme activity does not affect only the level of tryptamine. Aromatic L-amino acid decarboxylase deficiency (AADCD) manifests as a rare, autosomal recessive neurodevelopmental disorder characterized by impaired synthesis of crucial neurotransmitters such as dopamine, NE, adrenaline, and 5-HT [31] and trace amines such as tryptamine. This impairment results from biallelic mutations in the *DDC* gene. In contrast, an increase in AADC activity was observed in individuals with endogenous psychosis, such as schizophrenia [32].

In the gut, bacteria with AADC activity promote the decarboxylation of L-tryptophan to tryptamine. The gut symbionts *Clostridium sporogenes* and *Ruminococcus gnavus* are common components of the gut microbiota that generate tryptamine [33]. The metabolites produced by the gut microbiome impact host metabolism by controlling the release of gut hormones [34]. Given the heightened susceptibility of the serotonergic system to shifts in the early-life microbiome [35], the concentration of tryptamine is likely to exert a crucial influence on the development of neurodevelopmental disorders. Indeed, in a research in which the gut microbiota of human donors with ASD or typically developing controls was transplanted into germ-free (GF) mice, colonization with the microbiota of individuals with ASD alone was adequate to trigger characteristic autistic behaviours [36]. Additionally, the behavioural alterations induced by the microbiota of individuals with ASD were more prominent in male mice than in female mice [36]. Clarke and colleagues [37] demonstrated that male GF mice simultaneously exhibit a significant increase in plasma tryptophan concentrations and elevated levels of 5-HT and 5-hydroxyindoleacetic acid in the hippocampus. However, these changes are not observed in female GF mice [37]. The colonization of female GF animals by bacteria does not affect the elevation of 5-HT and 5-hydroxyindoleacetic acid levels in the hippocampus, but it does restore plasma tryptophan concentrations to the control level [37]. Importantly, the peripheral factor albumin regulates both brain and peripheral 5-HT levels, orchestrating interactions among the serotoninergic, immune, and endocrine systems [3]. In turn, lifestyle choices, environmental stimuli, and pharmacological interventions ultimately influence the regulation of neurogenesis in the hippocampus through their effects on the interactions mentioned above [4]. Therefore, in male GF mice, there is a decrease in brain-derived neurotrophic factor (BDNF) expression in the hippocampus, whereas no change is observed in female GF mice [37]. In contrast, Neufeld and colleagues reported elevated expression of BDNF and decreased expression of the 5-HT1A receptor gene in the hippocampal DG of female GF animals using *in situ* hybridization [38]. Interestingly, the intestinal expression of miR-200b and miR-200c depends on the microbiota that produces tryptamine [39]. The expression of miR-200b and miR-200c decreases during oral Listeria infection [40] and is increased by a mixture of Clostridia strains from the human microbiota [39]. Moreover, tryptamine stimulates enterochromaffin cells to release 5-HT, which in turn affects the activity of the enteric nervous system [20], often called the “second brain” due to its autonomy, thereby participating in microbiota-gut-brain communication. Hence, bacterially derived tryptamine appears to be an ideal candidate for regulating the gut-brain axis. However, prolonged exposure to tryptamine results in the depletion of endogenous 5-HT within the enteric nervous system [20], which invariably leads to pathological changes. On the basis of these reports, it seems evident that metabolites produced by gut bacteria do not regulate the gut-brain axis *via* a novel mechanism; instead, they promote an existing mechanism. However, the mechanism that they promote is unknown and will be clarified later.

Overall, tryptamine synthesis in both the gut and brain underscores its importance in various physiological and pathological processes. In the brain, tryptamine acts as a neuromodulator, influencing neurotransmitter systems and affecting mood and behaviour. In the gut, it is produced mainly by the microbial metabolism of tryptophan, which plays a crucial role in regulating gastrointestinal function and gut-brain communication.

## 3 DIFFERENCES IN TRYPTAMINE PHARMACOKINETICS BETWEEN THE BRAIN AND PERIPHERY

Tryptamine is lipophilic and hence able to cross cell membranes [41], including the blood-brain barrier (BBB) [42]. Following the intravenous administration of a nonconvulsant dose of tryptamine (5 mg/kg), the concentration of tryptamine in the brain increased to 0.173 ± 0.019 μg/g in just sixty to seventy-five seconds, demonstrating the ability of systemically administered tryptamine to cross the BBB. Additionally, its short endogenous half-life (less than 1 minute) within the brain [43], in contrast with its longer half-life in the blood (approximately 70 minutes) [44], implies that tryptamine synthesized in the brain may primarily impact nearby brain cells directly, whereas tryptamine originating outside the brain can also affect the brain.

Extensive research into brain regions with the highest tryptamine concentrations has validated a link between MAO activity and tryptamine levels. Generally, significant elevation of tryptamine levels (ng/g brain) has been documented in the whole brain following the administration of MAO inhibitors [45]. The administration of a nonselective and irreversible MAO inhibitor, tranylcypromine, resulted in elevated brain tryptophan levels, consequently leading to the accumulation of tryptamine. Furthermore, the tryptamine level after tranylcypromine treatment was three times greater than that following pargyline administration.

The most sensitive and specific techniques showed that the highest concentration of this trace amine was in the striatum and hippocampus (reviewed by Jones RS) [5]. In the striatum [46], the level of tryptamine significantly increased (up to 6 ng/g) 0.5 hours after the administration of at least 5 mg/kg clorgyline, a type A MAO (MAO-A) inhibitor. The tryptamine level subsequently remained relatively stable between 1 and 24 hours post-injection. Tryptamine concentrations significantly increased (up to 28 ng/g) only 1 hour after the administration of deprenyl (100 mg/kg), a selective irreversible inhibitor of type B MAO (MAO-B), and remained unaffected by the subsequent administration of this dose of deprenyl. Tryptamine concentrations peaked 4 hours after the administration of tranylcypromine (1 mg/kg), reaching 130 ng/g, before rapidly declining. Within 1 hour following the administration of phenelzine (100 mg/kg), an MAO inhibitor, a significant increase in tryptamine levels (up to 10 ng/g) was observed, and tryptamine concentrations increased for 6 hours before decreasing. Intraperitoneal injection of pargyline (75 mg/kg), which selectively inhibits MAO-B; iproniazyd (100 mg/kg), an irreversible inhibitor of MAO-A and MAO-B; and pheniprazine (15 mg/kg), an irreversible and nonselective MAO inhibitor, significantly increased tryptamine levels to 40 ng/g, 10 ng/g, and 20 ng/g, respectively, after 4 hours [46].

In the hippocampus, the brain region with the second highest concentration of tryptamine, a notable increase in tryptamine levels was demonstrated using mass spectrometry with the integrated-ion-current procedure. A remarkable 84-fold increase in tryptamine concentration was observed 1.5 hours after the tranylcypromine injection. Furthermore, these increases were significantly greater and more rapid than those in the concentrations of the putative neurotransmitter amines dopamine (DA), NA, and 5-HT under the same circumstances [47].

On the basis of Sullivan JP's findings [48], the highest intrinsic clearance (Vmax/Km) of tryptamine metabolism is calculated in the cerebral cortex and liver. The oxidation of tryptamine is carried out primarily by MAO-A in the liver, with MAO-A and MAO-B being responsible for comparable levels of tryptamine metabolism in the cerebral cortex. Subsequent research ruled out the participation of cytochrome P450 isoenzymes in tryptamine metabolism [49]. They confirmed this through experiments employing both human liver microsomes and microsomes expressing recombinant human MAO or P450 isozymes, revealing that human MAO-A is the primary mediator of the deamination of the trace amine tryptamine, whereas MAO-B assumes a secondary role in human liver microsomes [49].

In summary, the difference in tryptamine pharmacokinetics between the brain (CNS) and the remaining body (peripheral tissues and organs) primarily arises from organ-specific metabolism *via* different MAO isoforms. The rapid passage of tryptamine through the BBB, coupled with its rapid half-life in the brain in contrast to its comparatively slower degradation in the periphery, reaffirms the auxiliary role of peripheral tryptamine in physiological brain function.

## 4 A LINK BETWEEN MAO ACTIVITY AND TRYPTAMINE IN BOTH THE BRAIN AND PERIPHERY IN HEALTH AND DISEASE

MAO is an enzyme anchored to the outer mitochondrial membrane through a transmembrane helix located within the carboxyl-terminal domain that is significantly expressed in both the brain and peripheral organs. This enzyme exists in two isoforms, MAO-A and MAO-B, which are present in the early stages of neuronal development, coinciding with the significant division of the brain into distinct regions [50]. MAO-A and MAO-B, which share 70% amino acid sequence similarity, are encoded by two distinct genes located on the short arm of the X chromosome [51], which has great utility in regenerative medicine, considering that it seems to be easier to obtain genetically stable male iPSC lines than to obtain genetically stable female iPSC lines [52]. *In vivo* measurements of brain MAO-A activity in 38 healthy adult male volunteers with high and low MAO-A genotypes revealed that the MAO-A genotype had no effect on MAO-A activity [53]. However, a direct correlation exists between the frequency of maternal smoking during pregnancy and the extent of cognitive impairments linked to damage to the hippocampus [54], the brain region pivotal in ageing and neurocognitive function. Therefore, other factors exert a more significant influence on the modulation of brain MAO activity in the adult brain than the MAO-A genotype does. Positron emission tomography (PET) revealed a reduction in brain MAO-A levels among chronic cigarette smokers [55]. Moreover, MAO-B in the brain is inhibited by cigarette smoke [56]. This finding implies that in addition to its established tumour-stimulating effects, cigarette smoking might also trigger a neuroprotective mechanism in the brain associated with the inhibition of MAO activity. Thus, MAOs can undergo various levels of modification, which can impact their structure and activity. These modifications are particularly important in the context of developing an MAO-related neuroprotective strategy for treating neuropsychiatric disorders.

**Fig. (3):**
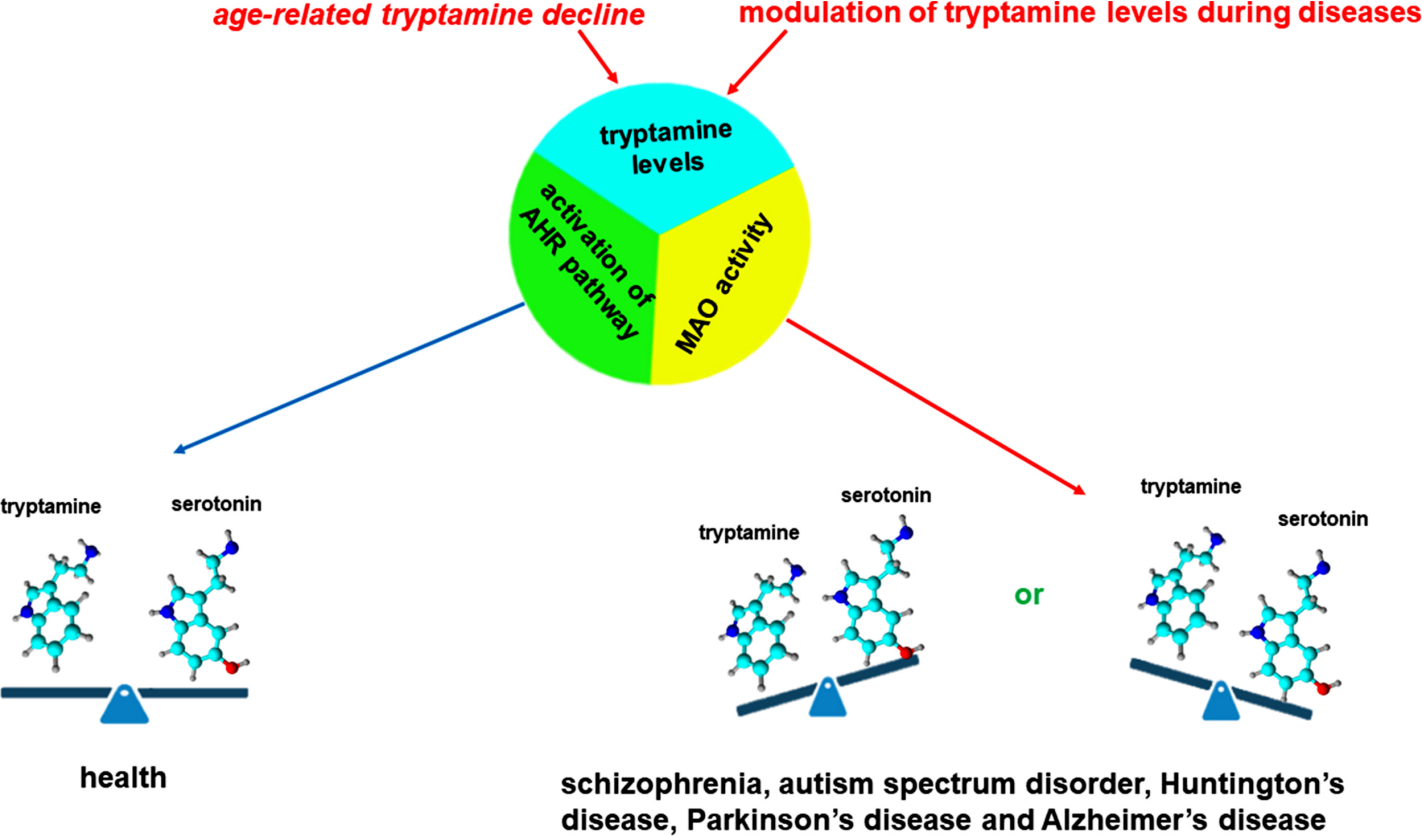
Relationships between tryptamine concentrations and serotonin concentrations.

In both the rat liver and brain, MAO-A precedes MAO-B during development, with a notable decrease in the average A:B ratio following birth, particularly in the brain [57]. This developmental pattern in the rat brain appears to parallel that in the human brain [58]. Interestingly, in the brain, the locus coeruleus, a pivotal site for neurogenesis, contains the highest concentration of MAO-A [59-61]. Additionally, elevated levels of 5-HT in the brains of MAO-A knockout pups induce cortical abnormalities, which can be reversed by administering PCPA, a 5-HT synthesis inhibitor [62]. Consequently, variations in the level of MAO-A during development could affect the physiological relationship between serotonergic system function and tryptamine levels in individuals with neurodevelopmental disorders (Fig. **3**).

Numerous MAO inhibitors appear to be effective treatments for various neurodegenerative diseases, such as PD [63] and AD [64]. It is widely recognized that reactive oxygen species (ROS), such as hydrogen peroxide (H_2_O_2_), generated by MAO affect both mitochondrial function and the cellular redox status [65]. Alterations in the cellular redox status impact numerous signalling cascades that regulate cellular proliferation and differentiation [66]. ROS derived from the endoplasmic reticulum (ER), including H_2_O_2_, inhibit cell growth and induce cell death and senescence [67, 68] in a manner that depends on neurodegenerative disease pathogenesis [69, 70]. The increased expression of miR-329, miR-193b, miR-20a, miR-296, and miR-130b in H_2_O_2_-stimulated primary hippocampal neurons derived from various strains of senescence-accelerated mice indicates the potential involvement of ROS-mediated modulation of miRNA expression in the pathogenesis of neurodegenerative diseases such as AD [71]. Conversely, decreased levels of miR-34b and miR-34c were observed in brain regions with diverse degrees of neuropathological alterations during the clinical (motor) stages of PD, the second most prevalent neurodegenerative disorder [72]. These regions include the amygdala, frontal cortex, substantia nigra, and cerebellum.

Both MAO-A and MAO-B are present in the human brain [73]. While MAO-A is predominantly found in noradrenergic neurons, moderately in serotoninergic neurons, and at very low levels in histaminergic neurons [61], MAO-B is most abundant in histaminergic and serotoninergic neurons [60, 61, 74]. Additionally, MAOs are present in nonmonoaminergic cell populations. Specifically, MAO-A is exclusively detected in neurons, such as hippocampal dopaminergic neurons, whereas MAO-B is highly expressed not only in neurons but also in nonneuronal cells, such as astrocytes [60, 74-76]. In addition, human platelets contain only MAO B [73].

When microtubule-associated protein 2 (MAP2, a marker of neuronal differentiation) is used as a reference gene, the expression levels of both MAO-A and MAO-B in the brains of patients with AD are 1.6 times greater than those in the brains of healthy subjects [77]. However, platelet MAO-B activity may increase in patients with AD [78, 79] and may remain unchanged [80] or be notably reduced in female patients in the late phase of AD compared with female patients in other phases of AD, as well as in healthy controls [81]. This inconsistency could be explained by variations in substrate (tryptamine) access by MAO and age discrepancies among the experimental groups. Substrate access by MAO both in the brain and in the periphery is regulated by albumin [3]. Furthermore, in patients with AD (age range: 49-73 years), MAO activity (nmol/min. per 10^9^ platelets ± S.E.M) was found to be greater (0.28 ± 0.04) than that in age- and sex-matched controls (0.20 ± 0.02) [78]. In another analysis, the mean MAO-B activity (nmol/h per 10^7^ platelets ± S.D.) in the different groups was as follows: control group (age range: 46-85 years) = 2.01 ± 0.73; untreated PD group (age range: 26-76 years) = 1.80 ± 0.54; levodopa-treated PD group (age range: 60-77 years) = 1.56 ± 0.55; and AD group (age range: 57-88 years) = 1.78 ± 0.87 [80]. The kinetics data for platelet MAO-B revealed increases in both Km and the maximum velocity (Vmax) in patients with AD [79]. These findings indicate that patients with AD require a relatively high concentration of MAO substrate to achieve half of the Vmax. This scenario arises when there is a low substrate concentration. This assessment suggests that patients with AD have a lower concentration of MAO substrate than needed. Additionally, the selectivity of MAO towards its substrate relies on the concentrations of the substrate and enzyme, as well as the substrate's affinity and turnover rate [82]. A substrate for both MAO-A and MAO-B is tryptamine [49]. *Ruminococcus gnavus*, a gram-positive anaerobic bacterium of the phylum *Firmicutes*, is a component of the gut microbiota and is known to produce tryptamine [83]. However, Mariat *et al*. reported that the *Firmicutes*/*Bacteroidetes* ratio significantly changes with age. Specifically, in infants (3 weeks to 10 months old), the ratio was 0.4; in adults (25 to 45 years old), it increased to 10.9; and in elderly individuals (70 to 90 years old), it decreased to 0.6 [84]. Thus, the natural decline in tryptamine availability with age contrasts with the increase in the need for tryptamine in individuals with AD. This implies the presence of a previously undiscovered neuroprotective mechanism of tryptamine in AD, achieved by the elevation of endogenous tryptamine levels. An unconventional question emerges: can the transplantation of the gut microbiota of healthy adults (up to 45 years old) into people with AD significantly alter the gut microbiota to produce more endogenous tryptamine? More research on this topic is needed; however, research based on animal models reveals that lower doses of psychedelic compounds such as psilocybin promote hippocampal neurogenesis, whereas higher doses suppress hippocampal neurogenesis and exert potent neuroprotective effects (reviewed by Vann Jones SA) [85]. Psilocybin is structurally similar to tryptamine and is metabolized in the body to produce psilocin, which is responsible for the psychoactive effects commonly associated with consuming magic mushrooms.

Overall, tryptamine is metabolized primarily by monoamine oxidase (MAO), which catalyses the oxidative deamination of tryptamine, converting it into indole-3-acetaldehyde. This metabolic pathway is crucial for regulating tryptamine levels and ensuring that its effects are transient. The precedence of MAO-A over MAO-B during development has significant consequences for neurotransmitter regulation, neurodevelopment, and the risk of neurodevelopmental and psychiatric disorders. In patients with Alzheimer's disease, the expression levels of both MAO-A and MAO-B in the brain are greater than those in healthy controls. Platelet MAO-B activity in these patients depends on tryptamine access by MAO and the natural decrease in tryptamine availability with age.

BBB leakage is prevalent in patients with dementia and in patients with AD, it may result from associated cerebrovascular pathology rather than solely from AD pathogenesis itself [86]. BBB dysfunction in AD can lead to increased transudation, allowing substances such as albumin to pass from the bloodstream into the brain's extracellular space [87]. In turn, extravasal albumin in the hippocampus induces rapid changes in astrocyte function [88]. As albumin participates in complex interactions among the serotoninergic, immune, and endocrine systems, misfolding of albumin can also lead to neuroinflammation in the brain [3, 4]. The activation of immune cells within the CNS, including microglia and astrocytes, along with the release of inflammatory mediators such as cytokines, chemokines, and ROS, is a common feature of neuroinflammation. This process contributes to the progression of diseases such as AD, PD, Huntington's disease, amyotrophic lateral sclerosis (ALS), and multiple sclerosis (MS) [89]. Moreover, astrocytosis is considered an early event in the progression of AD [90]. In astrocytosis, astrocytes become activated and undergo morphological and functional changes, such as increased expression of glial fibrillary acidic protein (GFAP) and secretion of proinflammatory cytokines. Additionally, in the brains of AD patients, increased regional binding of [11C]-l-deprenyl (an irreversible inhibitor of MAO-B) is positively associated with an increased number of activated astrocytes, which highly express MAO-B [91]. A critical defence mechanism against Aβ (β-amyloid)-induced neuroinflammatory responses in astrocytes is the activation of autophagy [92]. In turn, elevated levels of tryptamine (0.1-1 mM) induce autophagy in the HT22 and SK-N-SH nerve cell lines, as well as in primary cultured astrocytes [93]. These findings suggest that tryptamine exerts neuroprotective effects by suppressing neuroinflammatory responses *via* the enhancement of autophagy. Nevertheless, further research is needed on this topic.

## 5 TRYPTAMINE AS AN ENDOGENOUS AGONIST OF AHR

Tryptamine, an endogenous ligand of AHR, activates the AHR signalling pathway [94, 95]. Moreover, AHR activation is positively correlated with the activity of the MAO system [96]. Thus, the upregulation of CYP1A1 following AHR activation by tryptamine is directly linked to the activity of the mitochondrial enzyme MAO. However, tryptamine is the most critical component of the self-regulating AHR pathway, as tryptamine inhibits AHR target genes. Tryptamine has been shown to inhibit CYP1A1 and CYP1A2 [96, 97]. Therefore, tryptamine is involved in the precise regulation of AHR signalling pathway activation.

The AHR signalling pathway is pivotal for numerous physiological and pathological processes. The dysregulation of AHR signalling has been linked to a range of immune-related disorders. This connection arises from the complex function of AHR in sustaining immune system equilibrium and involves intricate interactions with immune cells, cytokines, and environmental elements [98, 99]. Moreover, AHR modulates the cellular signalling pathways essential for cell proliferation, differentiation [100] and apoptosis [101]. Nonetheless, the effects of AHR ligands on apoptosis vary significantly depending on the tissue and context. At times, AHR activation appears to have no effect on apoptosis rates [100], whereas at other times, it does [102, 103]. Additionally, there are critical developmental periods during which AHR-mediated changes in proliferation and differentiation occur [100]. Therefore, the interplay between AHR and the androgen receptor (AR), which is linked to dysfunction of the serotonergic system [3, 104-106], could contribute to the onset of neurodevelopmental disorders such as ASD. Furthermore, the pathological manifestation of this neurodevelopmental disorder may result from disturbances in the mechanisms governing the relationship between endogenous tryptamine levels and the serotonergic system.

Upon ligand binding, AHR typically undergoes a conformational change, allowing it to translocate from the cytosol to the nucleus, where it forms a heterodimeric complex with its partner protein aryl hydrocarbon receptor nuclear translocator (ARNT). This complex binds to specific DNA sequences within the regulatory regions of target genes; as a result, AHR regulates the expression of its target genes. However, ARNT also acts as a potent heterodimerization partner of oestrogen receptor alpha (ERα) and oestrogen receptor beta (ERβ)-dependent transcription and serves as an obligatory partner protein for conditionally regulated basic helix-loop-helix-PAS proteins such as HIF-1α [107]. Notably, the heterodimerization effects of ARNT on the ERβ are more pronounced than those on the ERα [108]. In female mice lacking functional ERβ due to ERβ knockout, regardless of whether circulating oestrogen is present in the plasma, increased anxiety and decreased levels of 5-HT or dopamine across different brain regions, including the striatum and hippocampus, are observed [109]. Anxiety is widespread among individuals with AD, is common in those with Huntington's disease, and is a prevalent nonmotor symptom of PD.

The use of psilocybin, which has structural similarities with tryptamine, appears to be linked to promoting smoking cessation [110]. Cigarette smoking alters epigenetic patterns by inducing DNA methylation changes that can be reversed upon smoking cessation, although specific genes remain differentially methylated up to 22 years after cessation [111]. Additionally, the methylation of the aryl hydrocarbon receptor repressor (AHRR) at cg0557592, which is strongly correlated with serum cotinine levels, can reliably predict cigarette smoking [112]. AHRR forms a heterodimer with AHR in the nucleus and participates in regulatory control over many AHR-responsive genes, including cytochrome P-450s and cyclooxygenase-2 (COX-2). One of the numerous effects facilitated by AHR *via* the induction of COX-2 [113] is the exacerbation of inflammation and the generation of Th17 cells associated with autoimmune conditions [114]. Furthermore, increased expression of the pro-oxidant enzyme COX-2 has been shown to be positively correlated with neurodegeneration in different pathological conditions, including PD [115].

In summary, the modulation of the AHR signalling pathway by tryptamine is pivotal for various physiological processes and may contribute to the development of neurodevelopmental disorders such as ASD and the progression of neurodegenerative diseases such as AD, Huntington's disease, and PD.

### 5.1 The Role of Hypoxia in the Activation of AHR

Cell differentiation is regulated by oxygen availability. Additionally, hypoxia can stimulate the development of inflammation [116], initiating a cascade of events that can result in hypoxia-induced inflammation and subsequent tissue damage or injury. However, under hypoxic conditions, there is a tendency for increased production of tryptamine and its subsequent metabolites [117]. Tryptamine treatment increases the mRNA expression levels of the AHR target, *TIPARP* [117]. *TIPARP* acts as a negative regulator of AHR by facilitating mono-ADP-ribosylation, thereby inhibiting its transcriptional activator activity. Generally, hypoxia-inducible factors (HIFs) are important transcriptional regulators of cell metabolism that facilitate adaptation to the cellular stress resulting from oxygen deficiency (hypoxia) [118]. Hypoxia (1% O_2_, 94% N_2_, and 5% CO_2_) leads to the stabilization of HIFs, primarily HIF-1α [119]. Following stabilization, HIF-1α translocates into the nucleus and regulates the expression of hypoxia target genes, either increasing or decreasing their expression. For example, severe hypoxia was shown to reduce albumin secretion, along with the upregulation of TGFβ family genes [120]. Notably, in the same report, albumin secretion was found to be approximately six times greater in the mild-hypoxia group (10% O_2_, using an O_2_-permeable plate) than in the excessive-hypoxia group (20% O_2_, using an O_2_-nonpermeable plate) and approximately two times greater in the mild-hypoxia group than in the ambient group (20% O_2_, using an O_2_-permeable plate) [120]. Because albumin coordinates interactions among the serotoninergic, immune, and endocrine systems, it may also control the regulation of AHR target genes [3]. Thus, when its levels are decreased, tryptamine seems to be a substitute for albumin in coordinating the regulation of AHR signalling under hypoxic conditions. Therefore, HIF-1α has been demonstrated to play dual roles, functioning either as a protective transcription factor or a proapoptotic factor depending on the severity of hypoxia and associated environmental conditions [121]. Consequently, efforts have been made to stabilize this protein in both neurodegenerative and psychiatric disorders [121, 122]. Notably, the involvement of HIF-1α, a redox-sensitive protein, in initiating early redox hypoxia-dependent processes during epithelial-mesenchymal transition (EMT) in cancer cells is also significant [123]. Additionally, the HIF-1α-dependent release of VEGF contributes to increased invasiveness during cancer progression and metastasis [123].

Overall, the regulation of AHR signalling under hypoxic conditions is controlled by tryptamine.

## 6 ARYL HYDROCARBON RECEPTOR-DEPENDENT INDUCTION OF CYP ENZYMES UNDER THE CONTROL OF miRNAs

**Fig. (4):**
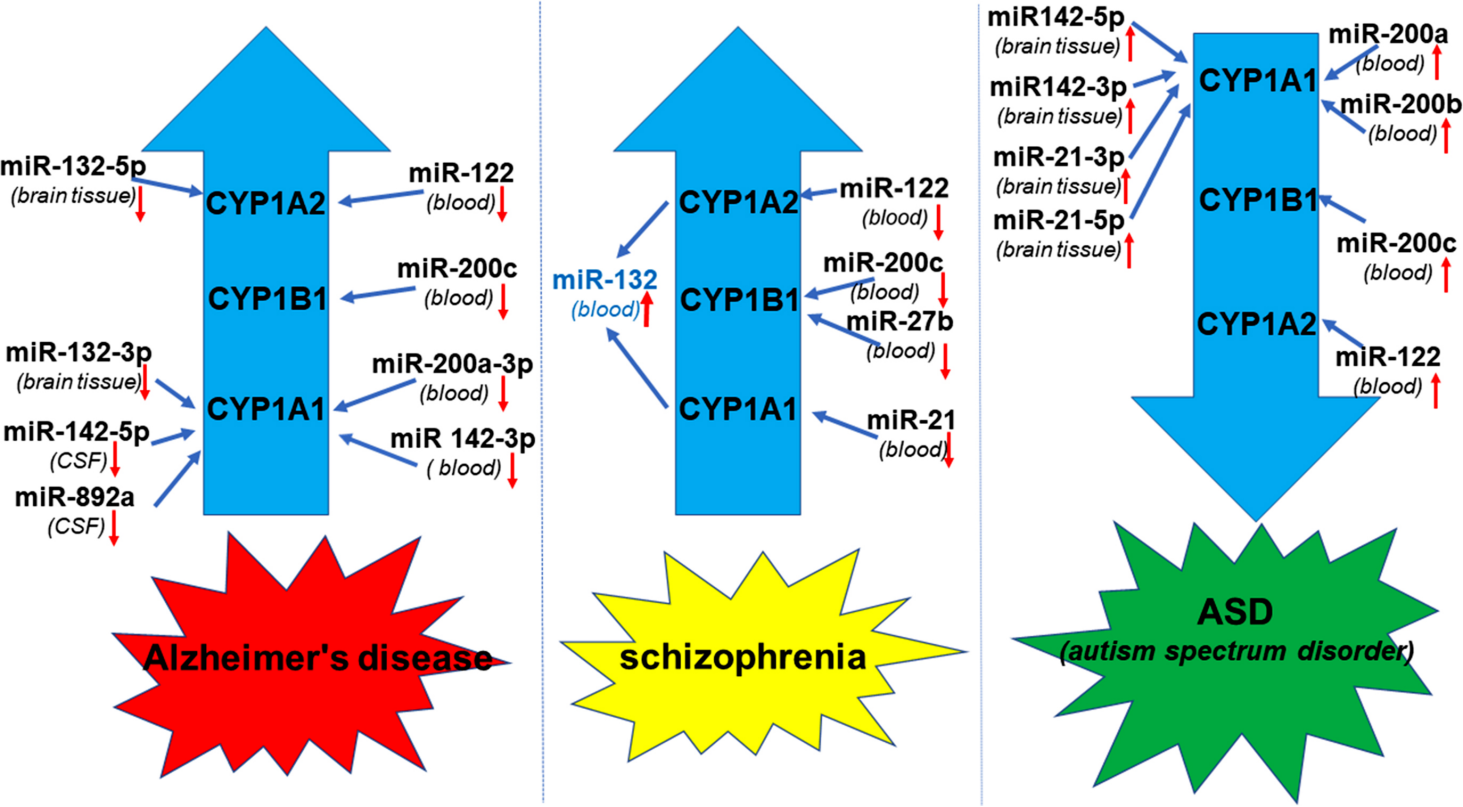
TheranoMiRNAs-CYP enzymes interconnection in Alzheimer’s disease (AD), schizophrenia and autism spectrum disorder (ASD).

The available literature has connected the dysregulation of miRNAs to the development of neurodevelopmental disorders such as ASD; psychiatric diseases such as schizophrenia; and the progression of neurodegenerative diseases such as AD or PD. On the basis of these reports, it is important to integrate the analysis of these miRNAs with the regulation of the enzymes CYP1A1, CYP1A2, and CYP1B1 to elucidate the functional associations between these miRNAs and CYP activity (Fig. **4**).

### 6.1 A Link to Neurodegenerative Diseases such as AD

A nearly perfect complementary sequence for miR-27b was identified in the 3'-untranslated region of human CYP1B1, indicating a significant inverse relationship between the miR-27b expression level and the CYP1B1 protein level [124]. Specifically, miR-27b expression was found to be reduced in breast cancer tissues, which corresponded with increased CYP1B1 protein levels. Additionally, miR-200c also directly targets CYP1B1, with an inverse correlation observed between miR-200c levels and CYP1B1 protein expression [125]. In renal cell cancer (RCC) tumours, low miR-200c expression is associated with elevated CYP1B1 protein levels [125].

CYP1B1 is known to regulate stem cell senescence and mitochondrial dysfunction, which are critical factors in neurodegenerative diseases [126]. Animal experiments have demonstrated that the upregulation of CYP1B1 is linked to the progression of tau pathology in the hTAU mouse model, which expresses six isoforms of human tau but is not associated with amyloid pathology progression in J20 mice [127]. Conversely, CYP1B1 deficiency has been shown to improve learning and memory deficits induced by a high-fat diet [128]. Analysis of brain tissue RNA-Seq data from the AMP-AD project revealed that CYP1B1 expression was elevated in the temporal cortex of Alzheimer's patients compared with that in cognitively normal older adults [127].

In short, CYP1B1 is upregulated in Alzheimer's disease. Epigenetic silencing of CYP1B1 may be caused by the upregulation of specific miRNAs.

Analysis of miRNAs revealed that miR-27b is upregulated in both the hippocampus and medial frontal gyrus of Alzheimer's disease patients [129]. However, a slight downregulation of miR-27b-3p was noted in the cerebrospinal fluid (CSF) of Alzheimer’s patients compared with healthy controls [130]. Dysregulation of miR-200c plays a role in the pathogenesis of Alzheimer’s disease, particularly in the context of the cognitive impairment associated with hyperphosphorylated tau [131]. Inhibiting miR-200c in the hippocampus of C57BL/6J mice has been shown to induce cognitive impairment and increase tau phosphorylation *via* the activation of 14-3-3γ. The upregulation of CYP1B1, which is linked to tau pathology progression in the hTAU mouse model expressing six isoforms of human tau, also contributes to disease development [127]. In the blood of Alzheimer’s disease patients, miR-200c is downregulated [131].

These analyses confirmed an inverse correlation between CYP1B1 and miR-200c, suggesting that this relationship could provide a viable strategy for diagnosing and treating degenerative diseases such as Alzheimer's disease.

An intriguing miRNA, miR-122 (miRNA-122), which is specifically conserved in the liver, regulates CYP1A2 expression *via* AHR [132]. Reduced exosomal expression of miR-122 in the blood of Alzheimer's disease patients [133] may lead to the derepression of CYP1A2 [132]. These findings suggest that increasing miR-122 levels and decreasing CYP1A2 activity could have therapeutic potential in Alzheimer's disease. This hypothesis is supported by reports showing that caffeine, which is metabolized primarily by CYP1A2 [134-136], can significantly protect against or reverse AD-like cognitive impairment and Aβ neuropathology in Alzheimer's disease mouse models [137]. Moreover, it seems that caffeine can occupy the active site of CYP1A2 and reduce the clearance of other substances that rely on CYP1A2 for metabolism. In turn, tryptamine may induce conformational changes in the CYP1A2 enzyme or alter its expression levels, thereby impacting its metabolic efficiency. Additionally, testosterone administration can induce miR-122 [138]. While several reports have linked low testosterone levels to an increased risk of developing Alzheimer's disease [139-141], elevated testosterone levels have also been associated with cognitive impairment [142].

A direct and specific interaction between miR-132-5p and the CYP1A2 3′-UTR has been demonstrated, indicating that miR-132-5p also inhibits CYP1A2 expression at the transcript, protein, and enzyme activity levels [143]. miRNA profiling of the human brain revealed that miR-132 is one of the most significantly downregulated miRNAs during the intermediate and late Braak stages of Alzheimer's disease, as well as in other neurodegenerative disorders [144-150]. Conversely, miR-132 levels are more than three times higher in patients with Parkinson's disease who are treated with dopamine receptor agonists than in healthy controls [145].

In short, CYP1A2 seems to be upregulated in Alzheimer's disease. Epigenetic silencing of CYP1A2 may be caused by the upregulation of specific miRNAs. Thus, the upregulation of miR-122 and/or miR-132-5p may be associated with decreased activity of CYP1A2 at the transcript, protein, and enzyme activity levels, which could have therapeutic potential in Alzheimer's disease.

The mRNA level of CYP1A1, a target gene of AHR, increased following Aβ treatment, but this effect was reversed by indoleamine 2,3-dioxygenase 1 (IDO1) inhibitors [151]. In Parkinson's disease, the inhibition rate of CYP1A1 was significantly lower in PD patients than in healthy controls (6.4% *vs*. 17.0% inhibition) [152], suggesting that altered CYP1A1 activity may be linked to the disease. Research on Alzheimer's disease revealed significant epigenetic changes in the CYP1A1 gene among AD patients [153]. Notably, both PD patients and AD patients showed significantly lower CYP1A1 inhibition rates than healthy controls [152, 153]. However, comparing PD and AD patients revealed significant differences in CYP1A1 inhibition rates, suggesting that distinct molecular mechanisms may be involved in transcript degradation or indirect transcriptional regulation of CYP1A1.

Microarray analysis revealed several miRNAs associated with reduced CYP1A1 gene expression, including miR-132, miR-142-3p, miR-200a, miR-200b, miR-21, and miR-34a [154]. Among these, the strongest negative correlations at the protein level were observed between CYP1A1 and miR-132, miR-142-3p, miR-150, miR-200a, miR-200b, miR-21, and miR-34a [154]. Additionally, research by Choi *et al*. demonstrated that miR-892a, which perfectly matches a sequence in the 3'-UTR of CYP1A1, inhibits CYP1A1 protein expression [155]. Furthermore, miR-892a was found to be less frequently detected in the cerebrospinal fluid (CSF) of symptomatic patients with sporadic frontotemporal dementia (FTD) and Alzheimer's disease (AD) than in presymptomatic patients and healthy controls [156].

Next-generation sequencing of the hippocampus of 41 late-onset Alzheimer's disease (LOAD) patients revealed significant downregulation of miR-132-3p and upregulation of miR-142-3p, miR-142-5p, and miR-200a-3p [144]. Real-time PCR confirmed the downregulation of miR-132-3p in the LOAD prefrontal cortex, hippocampus, and temporal gyrus [144]. This deregulation primarily occurs in neurons with Tau hyperphosphorylation [144].

MiR-142-5p showed opposite results, being either upregulated [130] or downregulated [129] in the cerebrospinal fluid (CSF) of patients with Alzheimer’s disease compared with healthy controls. Additionally, the downregulation of miR-142-3p was validated in the plasma of AD patients [157]. A slight downregulation of miR-21-5p was observed in the CSF of patients with Alzheimer's disease, with no change in miR-150-5p [130]. MicroRNA analysis revealed the upregulation of miR-34a in a double transgenic mouse model of Alzheimer's disease [158]. MiR-200a-3p is consistently downregulated in the brain tissue of AD mice, in cell culture models, and in the blood of AD patients [159].

In short, CYP1A1 appears to be induced in both Alzheimer's disease and Parkinson's disease. The epigenetic induction of CYP1A1 may be caused by the downregulation of specific miRNAs. Thus, upregulation of miR-132-3p, miR-892a, miR-142-3p, miR-142-5p and miR-200a-3p associated with silenced activity of CYP1A1 can have therapeutic potential in Alzheimer's disease.

In summary, the analysis above confirms that miRNAs are expressed in a tissue-specific manner, indicating that certain miRNAs may be abundant in brain tissue but present at much lower levels in blood or CSF. This specificity reflects the unique roles that miRNAs play in different biological contexts and cellular environments. The negative correlations of CYP1A1 with miR-132-3p, miR-892a, miR-142-3p, miR-142-5p and miR-200a-3p; CYP1A2 with miR-122 and miR-132-5p and CYP1B1 with miR-200c suggest direct regulation of CYP enzyme at the transcript, protein, and enzyme activity levels through transcript degradation or indirect transcriptional regulation. The above miRNA-CYP enzyme regulatory network analysis reveals a novel strategy for diagnosing and treating degenerative diseases such as Alzheimer's disease by verifying the interactions between miRNAs and CYP enzymes simultaneously. In particular, CYP activity can be easily and noninvasively detected in the blood *via* a metabolic cocktail. Moreover, miR-132-3p, miR-892a, miR-142-3p, miR-142-5p, miR-200a-3p, miR-122, miR-132-5p and miR-200c seem to be ideal theranoMiRNA in Alzheimer's disease.

### 6.2 A Link to Schizophrenia

Several reports suggest a possible link between CYP1A2 and schizophrenia or the response to antipsychotic drugs, although the evidence remains inconclusive. Approximately 18% of the antipsychotics used to treat schizophrenia, including clozapine, olanzapine, trifluoperazine and thiothixene are major substrates of CYP1A2 [160, 161]. In addition, cigarette smoking is particularly prevalent among individuals with psychotic disorders, with a rate of approximately 70-80% in those with schizophrenia [162]. While cigarette smoking may increase the risk of developing schizophrenia, it can also help alleviate certain symptoms, especially negative and cognitive symptoms [162, 163]. Additionally, cigarette smoking activates the aryl hydrocarbon receptor (AHR), which can lead to the upregulation of CYP1A1, CYP1A2, and CYP1B1. This activation can impact the metabolism of antipsychotic drugs. Pharmacokinetic analyses have shown that smokers have more rapid clearance of olanzapine and lower clozapine and norclozapine (desmethylclozapine) concentrations than nonsmokers [164]. CYP1A1 and CYP1B1 do not appear to be the primary enzymes responsible for the metabolism of these antipsychotic drugs. Therefore, further research is needed to clarify their role in the development and treatment of schizophrenia.

In short, all the CYP enzymes associated with the AHR receptor seem to be upregulated in patients with schizophrenia. Epigenetic induction of these CYP enzymes may be caused by the downregulation of specific miRNAs.

A significant increase in miR-132 expression was observed in the plasma samples of schizophrenia patients [165, 166]. Pharmacological treatment with olanzapine (which is metabolized primarily by CYP1A2) led to a notable decrease in miR-132 levels [167]. Improvements in clinical symptoms are closely associated with changes in miR-132 expression [167]. Conversely, miR-27b, miR-200c, and miR-21 have been reported to be downregulated in blood in several psychiatric disorders, including schizophrenia and major depressive episodes [168, 169]. Solexa sequencing and TaqMan low-density array (TLDA) assays revealed that the plasma levels of miR-122 were lower in schizophrenia patients than in control individuals [170]. In postmortem brain samples from schizophrenia patients, miR-27b, miR-34a and miR-132 were upregulated [171, 172]. Thus, this analysis also confirms the tissue-specific expression of miRNAs.

In summary, an inverse connection between CYP1A1 and miR-21; CYP1B1 with miR-27b and miR-200c; and CYP1A2 with miR-122 points to a miRNA-CYP enzyme regulatory network in schizophrenia that can be readily detected in the blood. Moreover, upregulation of miR-27b, miR-200c, miR-21 and miR-122 and downregulation of miR-132 associated with the silenced activity of CYP1A2 can have therapeutic potential in schizophrenia. Additionally, miR-27b, miR-200c, miR-21 and miR-122 and miR-132 may serve as ideal theranoMiRNAs in schizophrenia, particularly when analysed in conjunction with CYP1A2, CYP1B1 and CYP1A1 activities. Importantly, these analyses can be conducted noninvasively using patient blood. The aforementioned analysis of the miRNA-CYP enzyme regulatory network presents, for the first time, a strategy for diagnosing and treating schizophrenia through the simultaneous detection of miRNAs and CYP isoforms in blood.

### 6.3 A Link to Autism Spectrum Disorder (ASD)

An examination of prepubertal male children with ADHD revealed that those with higher autistic traits had significantly elevated serum testosterone and androstenedione levels than those with lower autistic traits [173]. Children with attention-deficit/hyperactivity disorder (ADHD) might have similar problems as those with autism spectrum disorder (ASD) and show impairments in social behaviour [173]. An increased level of testosterone in neurodevelopmental disorders may induce the expression of miR-122 in ASD. Additionally, the upregulation of miR-122 by testosterone could serve as a protective mechanism for melatonin, which is metabolized primarily by CYP1A2. However, these aspects remain unexplored and require further research. In particular, testosterone administration can increase miR-122 levels [138], potentially contributing to aggressive behaviour. In turn, CYP1A1, CYP1B1, and CYP1A2 are the main enzymes responsible for the 6-hydroxylation of melatonin, which is then conjugated with sulfate and excreted in the urine [174]. In addition, an abnormal melatonin circadian rhythm and below-average physiological levels of melatonin and/or its derivatives have been observed in individuals with ASD [175, 176].

CYP1A1 levels are reduced in the cord blood of individuals with ASD, and this gene has previously been shown to be transcriptionally regulated in blood by toxicants, such as polychlorinated biphenyls, that impact neurodevelopment [177-180]. El-Ansary and colleagues investigated the connection between CYP1B1-mediated vitamin D deficiency and autism and reported that plasma levels of CYP1B1 and vitamin D were 70% lower in children with autism than in age- and sex-matched neurotypical children [181].

In short, all the CYP enzymes associated with the AHR receptor are downregulated in ASD.

Epigenetic silencing of these CYP enzymes may be caused by the upregulation of specific miRNAs. In the hippocampi of offspring rats and humans, miR-134 and miR-132 levels are significantly elevated [182]. RNA sequencing of brain tissue from ASD autopsies revealed increased expression of miR-142-5p, miR-142-3p, miR-21-3p and miR-21-5p in Brodmann area 10, a region of the frontal cortex [183, 184], whereas miR-34a-5p was downregulated. Conversely, miR-34a was found to be upregulated in the brains of ASD model animals [185, 186]. In the blood of ASD patients, miRNA-27b and miR-142 were downregulated, whereas miRNA-200a, miRNA-200b, and miRNA-200c were upregulated [187].

Autism is a characteristic feature of X-linked MECP2 duplication syndrome in males [188]. Animal experiments have shown that reduced levels of miR-132 result in increased expression of MeCP2 [189].

In summary, an inverse correlation between CYP1A1, CYP1A2 and CYP1B1 was observed for the following miRNAs: (CYP1B1 with miR-200c; CYP1A2 with miR-122; CYP1A1 with miR-142-5p, miR-142-3p, miR-21-3p and miR-21-5p in brain tissue; and miRNA-200a and miRNA-200b in the blood of ASD patients. The above miRNA-CYP enzyme regulatory network analysis shows for the first time a strategy for the diagnosis and treatment of neurodevelopmental diseases such as autism spectrum disorder *via* the simultaneous detection of miRNAs and CYP isoforms. Analysis of miRNAs revealed that miR-142-5p, miR-142-3p, miR-21-3p and miR-21-5p are ideal theranoMiRNAs in the brain tissue of ASD patients, whereas miR-122, miR-200c, miRNA-200a and miRNA-200b in the blood of ASD patients.

## 7 CONSEQUENCES OF ALTERATIONS IN THE MECHANISMS GOVERNING ENDOGENOUS TRYPTAMINE LEVELS

The low physiological levels of tryptamine in the brain do not hinder its function in brain structures. Pathological manifestations occur when the mechanisms regulating tryptamine levels, particularly in brain regions with the highest concentrations of tryptamine, are disrupted. This phenomenon is evident in psychiatric, neurodevelopmental, and neurodegenerative disorders (Fig. **3**).

Compared with their mentally stable counterparts, chronic schizophrenia patients showed an approximately 50% increase in tryptamine levels in whole blood. This increase in blood tryptamine levels was positively correlated with a longer half-life of this trace amine, which was 120 minutes in schizophrenic patients and 70 minutes in healthy controls [44]; this phenomenon is linked to reduced activity of MAO [190] and cigarette smoking [191]. An epidemiological research revealed that 79% of chronic schizophrenic patients were smokers [192]. Among them, male schizophrenic patients had the highest smoking frequency at 93% (N=140 out of 150), followed by male nonschizophrenic patients at 78% (N=58 out of 74), female schizophrenic patients at 70% (N=61 out of 87), and female nonschizophrenic patients at 51% (N=25 out of 49) [192].

The abundance of species of the *Clostridium* genus is approximately tenfold greater in faecal samples from autistic children than in those from nonautistic children [193, 194]. The metabolic activity of specific species of the *Clostridium* genus is linked to the synthesis of tryptamine. However, this direct association between microbes and disease was not confirmed in another report. No notable variance was observed in the diversity or overall microbial composition between children with ASD and neurotypical (NT) siblings [195]. These findings suggest that the pathological manifestation of this neurodevelopmental disorder arises from the disturbance of mechanisms governing the level of endogenous tryptamine rather than the tryptamine level per se. One of these mechanisms could be associated with the interplay between AHR and AR, which is linked to dysfunction of the serotonergic system in the brain and peripherally [3, 104-106]. Variations in the prevalence of ASD between males and females appear to support this idea [196]. Thus, ASD results from a complex interplay of genetic, environmental, and possibly hormonal factors.

Alterations in neurogenesis are observed in various neurodegenerative disorders, including AD, PD, and Huntington's disease. GF mice exhibit increased hippocampal neurogenesis in adulthood, with a notable increase in the survival of newborn neurons [197]. This phenomenon is primarily observed in the dorsal hippocampus, which is known to be pivotally involved in spatial learning and memory [197]. However, the impact of the microbiota on hippocampal morphology and ultrastructure in GF mice appears to be associated with maladaptive changes in the hippocampus [198]. Volumetric analysis revealed hippocampal enlargement in GF mice. The ventral hippocampal pyramidal neurons in the GF mice were shorter, with fewer branches and spines. Dentate granule cells were less branched but the spine density remained unchanged in GF mice compared with controls [198]. After GF mice were colonized with the human gut microbiota, a significant increase in the tryptamine concentration in the faeces was observed [199]. Specifically, transplantation of the faecal microbiota of wild-type mice into mice with Huntington's disease positively influenced cognitive function in females [200]. However, in male mouse models of Huntington's disease, faecal microbiota transplantation (FMT) was ineffective [200]. An experiment involving stress-sensitive F344 male rats revealed that GF rats have lower concentrations of 5-HT in the hippocampus and higher concentrations of the dopamine (DA) metabolite homovanillic acid (HVA) in the striatum [37, 201]. However, they displayed a comparable stress-induced increase in both 5-HT and 5-HIAA levels as rats were exposed to a standard environment with normal microbiota [201]. The motor symptoms of PD stem from marked degeneration of dopaminergic neurons and their projections to the striatum [202]. The increase in striatal 5-HT levels observed in PD patients likely stems from a neural mechanism aimed at compensating for dopaminergic denervation, whereas the significant atrophy of the striatum observed in Huntington's disease patients may be correlated with the increase in 5-HT innervation [203]. Postmortem analyses have confirmed pathological changes in the shape of the striatum, one of the brain regions with the highest concentration of tryptamine, among AD patients; these changes were found to be positively associated with cognitive decline in these patients [204].

The consequences of alterations in the mechanisms regulating tryptamine levels are not always pathological. Higher concentrations of tryptamine exhibit anticancer (antiproliferative) properties. Tryptamine effectively inhibits HeLa cell growth by competitively inhibiting tryptophanyl-tRNA synthetase, leading to the consequent inhibition of protein biosynthesis [205]. At a concentration of 330 µg/ml, 70% of resistant HeLa A cells and 99.4% of control cells were killed with tryptamine. Compared with the control treatment, treatment with 100 μM tryptamine for 72 hours inhibited the growth of Jurkat cells (1.5x10^5^ cells/mL), resulting in a 70% reduction in growth [206]. In turn, ultrastructural analysis revealed that mouse HT22 and human SK-N-SH neuronal cells, as well as primary cultured astrocytes, exhibited autophagic features following a 24-hour incubation with 1 mM tryptamine [93]. Autophagy, which is also observed in various neurodegenerative disorders, is responsible for the degradation and recycling of damaged or unnecessary components within cells. Postmortem analyses of the brains of PD patients revealed apoptosis and autophagy in melanized neurons of the substantia nigra [207]. These neurons are part of the dopaminergic system in the brain, and they produce and release the neurotransmitter dopamine. In Huntington's disease, the stimulation of dopamine-mediated autophagy and degeneration in striatal neurons are also observed [208]. Brain biopsies from patients with AD unequivocally identified autophagosomes and other prelysosomal autophagic vacuoles (AVs) within neuritic processes, including synaptic terminals [209]. These reports demonstrate that autophagy plays a role in both neurodegenerative and regenerative processes in neurodegenerative disorders. Therefore, disruption of the mechanisms regulating both neurodegenerative and regenerative processes linked to neurodegenerative disorders could stem from disruption of the relationship between endogenous tryptamine levels and serotonergic system function.

In conclusion, the effects of tryptamine can be both beneficial and detrimental, depending on the context and concentration. Owing to this dual role, tryptamine acts as a double-edged sword in the search for therapeutic strategies for neurodegenerative, neurodevelopmental, and psychiatric disorders. Additionally, the influence of tryptamine appears to be significantly shaped by patient sex.

## 8 DYSREGULATION OF THE MECHANISMS CONTROLLING PROLACTIN LEVELS BY TRYPTAMINE

**Fig. (5):**
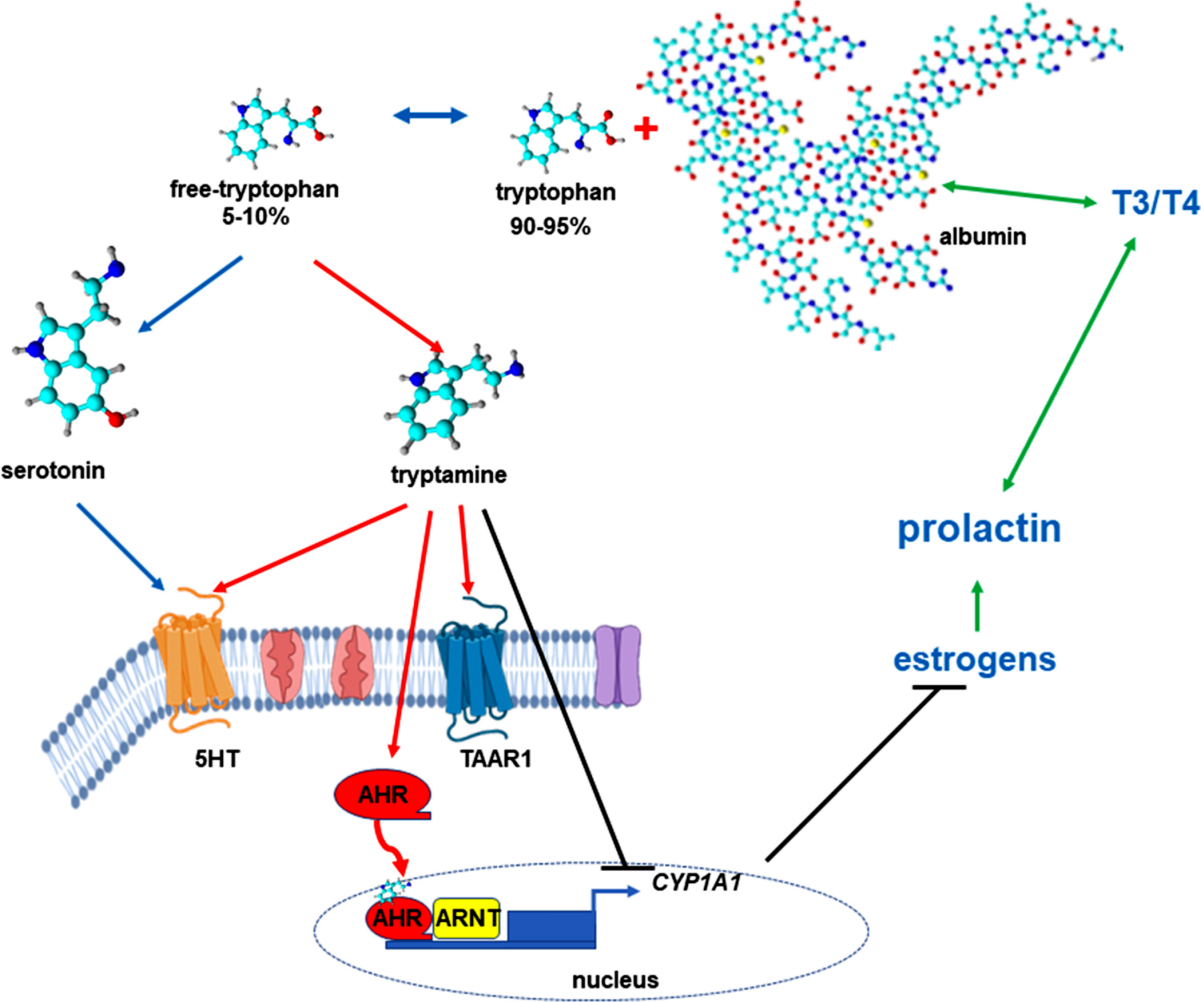
Role of tryptamine in regulating prolactin levels.

The tryptamine concentration steadily increases throughout the day and further increases at night. According to measurements of the average log10 (urinary indoleacetic acid (IAA)/tryptamine (T)) ratio, tryptamine metabolism is reduced during nocturnal sleep [210]. The circadian rhythm affects the transcription of MAO-A, as the transcription of the MAO-A promoter is governed by the clock components BMAL1, NPAS2, and PER2 [211]. One of the initial outcomes of AHR activation is the attenuation of the immunomodulatory effects of prolactin [212]. The activation of AHR impacts the circadian rhythm of serum prolactin (PRL) levels, establishing a connection between early alterations in the level of PRL-an essential immunomodulatory hormone-and subsequent changes in serum T4 and corticosterone concentrations [212, 213]. Thyroid hormones can either enhance or suppress the effects of PRL, depending on the context. T3 can specifically inhibit PRL synthesis and decrease PRL mRNA levels in cultured pituitary cells [214]. In a previous analysis, serum T_4_ concentrations were found to be significantly reduced in the hyperprolactinaemic group [215]. Furthermore, AHR activation is significantly associated with the inhibition of the interaction between thyroxine and albumin, which binds tryptophan [3, 104], the precursor of tryptamine (Fig. **5**).

In addition to thyroid hormones, oestrogens can influence the secretion of PRL [216]. Oestrogens, a group of steroid hormones, primarily ostradiol, estrone, and estriol, which are produced primarily in the ovaries (in females) and in smaller amounts in the testes (in males) and adrenal glands, exert their effects by binding to oestrogen receptors (ERs), including ERα and ERβ, which are present in various tissues throughout the body. However, through physical interactions between AHR and ERα, the degradation of ERα is facilitated after the activation of AHR [102, 217]. AHR ligands induce proteasome-dependent degradation of the ERα protein, a process that is absent in benzo(a)pyrene-resistant MCF-7 cells lacking AHR expression [102, 217]. This effect appears to be significant because oestrogen induces biphasic stimulation of PRL gene transcription [218]. Specifically, the transcription of PRL is first stimulated within 30 minutes after oestrogen treatment, and this effect persists for up to 2 hours after treatment. Additionally, a delayed phase of transcriptional stimulation is observed at 6 hours following oestrogen treatment, persisting for up to 24 hours. The reduction in ERα expression due to ERα knockdown confirms the significant role of ERα in AHR agonist-induced CYP1A1 expression, although no decrease in induced CYP1B1 expression was detected [219]. Furthermore, both CYP1B1 and CYP1A1 participate in oestrogen synthesis and metabolism, exhibiting differing degrees of catalytic activity and distinct regioselectivity [220].

A meta-analysis of PRL blood concentrations revealed elevated levels in antipsychotic-naïve patients experiencing a first episode of psychosis (AN-FEP), indicating that PRL is a potential biomarker for this illness [221]. A positive correlation exists between psychiatric symptoms observed in psychiatric patients and fluctuations in urinary tryptamine levels [222]. Specifically, a higher urinary tryptamine concentration appears to be linked to an increase in psychotic behaviour in schizophrenic patients [223].

A consistent association between PRL levels and urinary tryptamine levels was also observed in patients with the neurodegenerative disease PD. Compared with age-matched controls, patients diagnosed with PD showed elevated blood PRL levels [224-226]. Moreover, among males with PD, PRL levels were negatively correlated with sex steroid concentrations [226]. Additionally, increased levels of sex hormones were linked to improved mood and quality of life in males with PD [226]. Analysis of urinary tryptamine levels confirmed that patients with PD excrete higher concentrations of tryptamine through the urine [227].

In summary, the activation of AHR signalling through tryptamine binding leads to the dysregulation of mechanisms controlling PRL levels, which could play a pivotal role in psychiatric and neurodegenerative disorders.

## CONCLUSION

The therapeutic implications of the relationship between tryptamine levels, MAO activity, and AHR signalling pathway activation described here lies in its ability to slow down the neurodegeneration process and exert neuroprotective effects in the brain and periphery in neurodegenerative, neurodevelopmental, and psychiatric disorders.

In neurodegenerative disorders, such as Alzheimer's disease and Parkinson's disease, an increase in tryptamine levels, stimulation of AHR, along with concomitant inhibition of the enzymes CYP1A1, CYP1B1 and CYP1A2, and inhibition of MAO could improve neurotransmitter balance and reduce neuroinflammation, potentially slowing disease progression.

In psychiatric disorders such as schizophrenia, decreasing tryptamine levels by reducing AADC activity, stimulating AHR signalling, and carefully inhibiting MAO activity may improve neurotransmitter balance, reduce neuroinflammation, and enhance cognitive function, potentially providing new treatment options for this complex disorder.

In neurodevelopmental disorders such as autism spectrum disorder (ASD), where neurotransmitter dysregulation is prevalent, targeting the tryptamine pathway and AHR signalling could present new treatment opportunities. In particular, the modulation of the AHR signalling pathway by tryptamine and the dominance of MAO-A over MAO-B during development may impact the inverse relationship between endogenous tryptamine levels and serotonin levels, potentially contributing to the development of neurodevelopmental disorders such as ASD. Moreover, it can be hypothesized that the interaction of AHR with oestrogen receptors and the presence of MAO on the X chromosome contribute to the sex differences observed in autism spectrum disorder. The presence of two X chromosomes and the interaction between AHR receptors and oestrogen receptors (ERs) enable girls with autism to mask their symptoms (camouflage).

Moreover, the miRNA-CYP enzyme regulatory network analysis in this review paper presents for the first time a strategy for the diagnosis and treatment of neurodegenerative, neurodevelopmental, and psychiatric disorders *via* the simultaneous verification and regulation of two different functional partners, miRNA and CYP enzymes. The identification of the following theranomicroRNAs: miR-132-3p, miR-892a, miR-142-3p, miR-142-5p, miR-200a-3p, miR-122, miR-132-5p and miR-200c in Alzheimer's disease patients; miR-27b, miR-200c, miR-21 and miR-122 and miR-132 in schizophrenia patients; miR-142-5p, miR-142-3p, miR-21-3p and miR-21-3p in the brain tissue of ASD patients, and miR-122, miR-200c, miRNA-200a and miRNA-200b in the blood of ASD patients will aid in the development of therapeutic strategies for Alzheimer’s disease (AD), schizophrenia and autism spectrum disorder (ASD), particularly when analysed in conjunction with CYP1A2, CYP1B1 and CYP1A1 activities.

Overall, it is crucial to recognize that only the simultaneous modulation of tryptamine levels, MAO activity, and AHR signalling pathway activation has therapeutic potential for each pathological condition.

## LIST OF ABBREVIATIONS

AADC: Aromatic Amino Acid Decarboxylase
AHR: Aryl Hydrocarbon Receptor
AHRR: Aryl Hydrocarbon Receptor Repressor
ARNT: Aryl Hydrocarbon Receptor Nuclear Translocator
AD: Alzheimer’s Disease
ALS: Amyotrophic Lateral Sclerosis
AR: Androgen Receptor
ASD: Autism Spectrum Disorder
AV: Autophagic Vacuole
BBB: Blood-brain Barrier
BDNF: Brain-derived Neurotrophic Factor
CNS: Central Nervous System
COX: 2-cyclooxygenase-2
CYP: Cytochrome P-450 Isoform
cAMP: Cyclic Adenosine Monophosphate
DA: Dopamine
EC50: Half Maximal Effective Concentration
ER: Oestrogen Receptor
GF: Germ-free
GFAP: Glial Fibrillary Acidic Protein
HAS: Human Serum Albumin
HIF: Hypoxia-inducible Factor
HVA: Homovanillic Acid
Km: Michaelis Constant
MAP2: Microtubule-associated Protein 2
MAO: Monoamine Oxidase
MAO-A: Monoamine Oxidases A
MAO-B: Monoamine Oxidases B
MDMA: 3,4-Methylenedioxymethamphetamine, “ecstasy”
MS: Multiple Sclerosis
NE: Noradrenaline
PCA: p-chloroamphetamine
PCPA: p-chlorophenylalanine
PD: Parkinson’s Disease
PNS: Peripheral Nervous System
PRL: Prolactin
PTSD: Post-traumatic Stress Disorder
5-HT: Serotonin, 5-hydroxytryptamine
ROS: Reactive Oxygen Species
TAAR1: Trace Amine Associated Receptor 1
TheranoMiRNA: Therapeutic miRNA
Vmax: Maximum Velocity
